# Vibro-Perception of Optical Bio-Inspired Fiber-Skin

**DOI:** 10.3390/s18051531

**Published:** 2018-05-12

**Authors:** Tao Li, Sheng Zhang, Guo-Wei Lu, Yuta Sunami

**Affiliations:** 1 Institute of Innovative Science and Technology, Tokai University, Hiratsuka-shi 259-1292, Japan; taoli@tsc.u-tokai.ac.jp; 2 Micro/Nano Technology Center, Tokai University, Hiratsuka-shi 259-1292, Japan; sunami@tokai-u.jp; 3 Department of Mechanical Engineering, Tokai University, Hiratsuka-shi 259-1292, Japan

**Keywords:** fiber-skin, bio-inspired sensor, silicone elastomer, vibro-perception, OPLL

## Abstract

In this research, based on the principle of optical interferometry, the Mach-Zehnder and Optical Phase-locked Loop (OPLL) vibro-perception systems of bio-inspired fiber-skin are designed to mimic the tactile perception of human skin. The fiber-skin is made of the optical fiber embedded in the silicone elastomer. The optical fiber is an instinctive and alternative sensor for tactile perception with high sensitivity and reliability, also low cost and susceptibility to the magnetic interference. The silicone elastomer serves as a substrate with high flexibility and biocompatibility, and the optical fiber core serves as the vibro-perception sensor to detect physical motions like tapping and sliding. According to the experimental results, the designed optical fiber-skin demonstrates the ability to detect the physical motions like tapping and sliding in both the Mach-Zehnder and OPLL vibro-perception systems. For direct contact condition, the OPLL vibro-perception system shows better performance compared with the Mach-Zehnder vibro-perception system. However, the Mach-Zehnder vibro-perception system is preferable to the OPLL system in the indirect contact experiment. In summary, the fiber-skin is validated to have light touch character and excellent repeatability, which is highly-suitable for skin-mimic sensing.

## 1. Introduction

Human skin is the largest sensory organ, covering more than 90% of the entire body. In order to perceive the counter-surface, a wide variety of sensory neuron subtypes are innervated in the bio-skin, including low-threshold mechanoreceptors (LTMRs) [1]. When the skin slides over the surface of an object, the LTMRs encode mechanical stimuli by detecting the variation of stresses and the magnitude of vibrations [2]. Such detection helps skin to distinguish the roughness, stickiness, temperature, comfort level, etc., through the somatosensory system [3]. This unique and complex ability of tactile perception attracts considerable attentions in the robotics field [4]. Researchers believe that combining the perception-induced sensing modalities to robotics can provide better manipulation, exploration and enhance the physical interactions with objects similar to human manipulation [5].

To achieve this goal, sensor devices towards an efficient skin perception system need to be developed as tools implemented in humanoids. At present, the most popular sensors used in the e-skin are based on piezo-electrical components with different piezo-materials. Many researchers have started to develop the e-skin using different piezo-electrical materials such as hollow nanospheres exhibiting the sensitivity of 31.6 kPa${}^{-1}$[6], gelatin nanofibers with low piezo–electricity ($d_{33}\approx 20$ pm/V) [7,8], mesoporous ferroelectric poly (vinylidene-fluoride) films with the storage capacity of 0.118 μAh within 240 s [9], and nanotube films with conductivities as high as 2200 S·cm${}^{-1}$ [10]. In addition, the artificial skin based on optoelectronic technology has been proposed by using infrared Light Emitting Diodes (LED) to sense mechanical deformation [11], but exhibits high power consumption. Compared with the traditional e-skin, the self-powering e-skin can sense the sweat metabolites, electrolytes and skin temperature without the bulky electrical power [12,13,14]. Zhou et al. has proposed a tubular-shaped e-skin array to measure the bending distortion [15]. The piezoresistive interlocked microdome arrays-based e-skin have proposed for the pressure and temperature discrimination in [16,17]. Despite the above mentioned functions of the proposed e-skin, it is susceptible to the electromagnetic interference.

The standard single-mode optical fiber, an alternative sensor of tactile perception, is characterized by high sensitivity and reliability, low cost and low susceptibility to magnetic interference. Certain applications can be used for the low-loss micro/nano scale optical waveguides, evanescent wave sensors and waveguide couplers [18,19,20]. In particular, an optical interferometer has the capability to detect vibrations [21,22,23], sense angular rates [24,25], and measure temperature [26,27,28], etc. Due to the advantages of micro/nano technology, the size of optical fibers can be reached in micro/nano scale as small as 20 nm for the sensor application [29]. The light energy loss from optical fiber is extremely low, rendering it highly suitable as a micro-sensor device [30]. In this paper, the bio-inspired fiber-skin is designed as a micro-sensor with minimal energy losses to detect physical motions (tapping and sliding) as the human skin.

## 2. Optical Fiber-Skin Perception System

In this section, the details of the bio-inspired fiber-skin are introduced, along with the proposed methods for the vibro-perception of the fiber-skin: Mach-Zehnder and Optical Phase-Locked Loop (OPLL) vibro-perception system. Based on control theory, both the open-loop (Mach-Zehnder) and closed-loop (OPLL) fiber-skin perception experiments are conducted.

### 2.1. Bio-Inspired Optical Fiber-Skin

**Figure 1:**
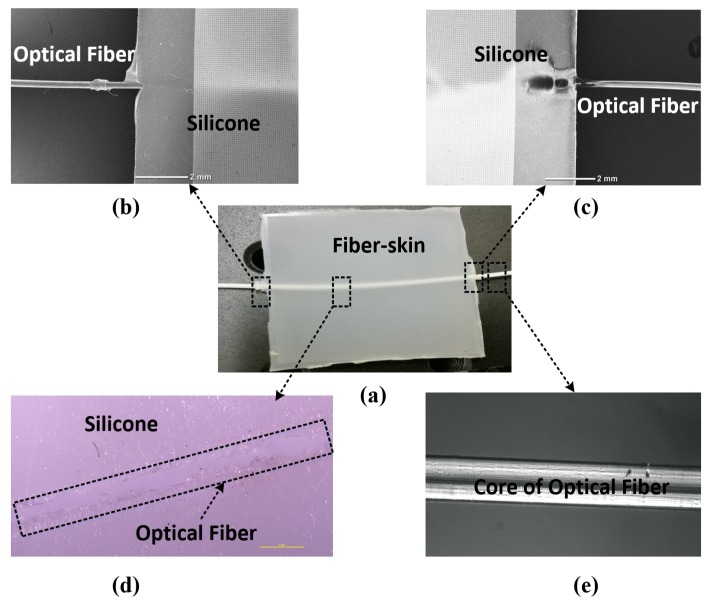
Sample of vibro-perception fiber-skin. (**a**) Physical sample of fiber-skin; (**b**) SEM (JCM-6000PLUS, JEOL Ltd.) image of the connecting port (right); (**c**) image of the connecting port (left); (**d**) embedded area of optical fiber; (**e**) optical fiber core.

The fiber-skin uses an optical fiber as the sensor to mimic cutaneous receptor of the human skin in form of vibration. Cutaneous receptors are the most important mechanoreceptors which are served as a tactile sensor to detect intensity of perceptual stimulations, e.g., vibration, pressure, touch, etc. [31]. Figure 1a,d give the prepared fiber-skin sample with optical fiber embedded in silicone elastomer.

In order to test the performance of the fiber-skin, connecting ports are linked to the Mach-Zehnder and OPLL systems which are specially designed to mimic the tactile sensations as human skin towards vibration (refer to Figure 1b,c). As shown in Figure 1e, the chosen optical fiber (Haphit Ltd. 201408-G652D) is made of silica material which possesses high flexibility and transparency. The fiber core radius is approximately 100 μm. The properties of intensity, phase, polarization, and wavelength for light propagation in the optical fiber grant the ability to measure strain, temperature, pressure, especially vibration.

The substrate material is silicone elastomer (HTV-2000, Prosilicone). According to the literature, silicone material has high biocompatibility for biomedical application such as surgical implantation, biomedical devices, dynamic bioreactors, etc. [32]. Furthermore, silicones demonstrate great coupling efficiency and tolerance of temperature and humidity, making it highly-suitable for attractive actuator like artificial muscle, skin, etc. [33]. In addition, silicone elastomer is highly adhesive with a low transition temperature, rendering it an ideal substrate for optical fiber insertion towards the fiber-skin.

### 2.2. Mach-Zehnder Vibro-Perception Perception of Optical Fiber-Skin

**Figure 2:**
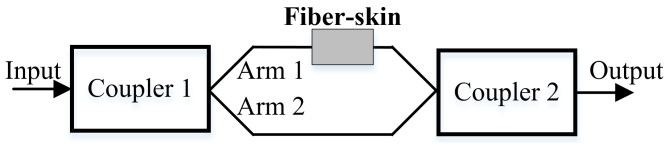
Schematic diagram of Mach-Zehnder vibro-perception system. The input is the optical signal from the laser. The coupler 1 and coupler 2 are 3 dB couplers that are used to construct the Mach-Zehnder interferometer.

According to the principle of the Mach-Zehnder interferometry, we propose the Mach-Zehnder vibro-perception system. Figure 2 shows the schematic diagram of the Mach-Zehnder vibro-perception system that can detect the phase errors introduced in the two arms. Therefore, once the designed fiber-skin is touched in the located arm, we can observe the perception results from the output port.

Supposing that the signals within Arm 1 and Arm 2 have the forms of $E_{1}e^{i(\phi_{1}-\omega t)}$ and $E_{2}e^{i(\phi_{2}-\omega t)}$, the beating result from the coupler 2 is expressed as the superimposition between them [34]:

(1) $$E_{s}=E_{1}e^{i(\phi_{1}-\omega t)}+E_{2}e^{i(\phi_{2}-\omega t)}$$

where $E_{1}$ and $E_{2}$ are complex magnitudes of the optical field within Arm 1 and Arm 2 accordingly. $\phi_{1}$ and $\phi_{2}$ are the phases of optical signals within Arm 1 and Arm 2. ω and *t* are the optical frequency and time, respectively. The phase errors between the two arms are tracked by measuring the interference intensity ($I_{s}$). The following expression shows that the interference intensity (or beating intensity) exhibits a proportional relation with the magnitude of the optical signals:

(2) $$I_{s}\;\propto \;{E_{1}}^{2}+{E_{2}}^{2}+2E_{1}E_{2}cos\left(\phi_{1}-\phi_{2}\right)$$

For the Mach-Zehnder fiber-skin perception system, the intensities in Arm 1 ($I_{1}$) and Arm 2 ($I_{2}$) are identical, therefore, the local intensities are symbolized as *I*. The above equation is modified as follows:

(3) $$\begin{array}{ccc} I_{s} & \propto & I_{1}+I_{2}+2\sqrt{I_{1}I_{2}}cos\left(\phi_{1}-\phi_{2}\right)\\ & \propto & I\left[1+cos\left(\phi_{1}-\phi_{2}\right)\right] \end{array}$$

Equation (3) shows that the detected signal of the Mach-Zehnder fiber-skin perception system behaves as a cosine wave, and the interference intensity will change according to the phase difference when we touch the fiber-skin.

### 2.3. OPLL Vibro-Perception System of Optical Fiber-Skin

The perceptual signals are difficult to obtain due to the effects of external disturbances from tapping and sliding motions on the interference intensity. Therefore, the OPLL vibro-perception system is designed to eliminate this problem. The main idea of OPLL vibro-perception is to keep the phase identical between Arm 1 and Arm 2. In this case, the interference intensity of the optical field is only proportional to the local intensity, namely, $I_{s}\;\propto \;I$.

**Figure 3:**
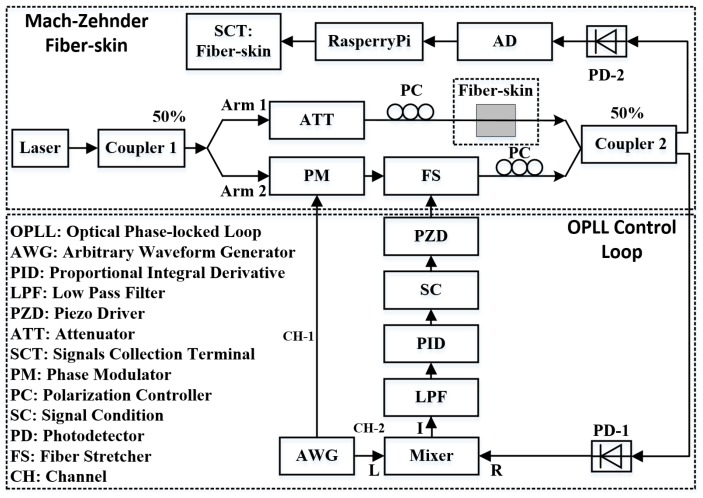
Schematic diagram of the OPLL fiber-skin perception system. The symbols *I*, *L* and *R* around the mixer present intermediate frequency, local oscillator and RF respectively.

The OPLL is comprised of the phase detector, the loop filter and the voltage-controlled oscillator (VCO). As introduced in Section 2.2, the Mach-Zehnder vibro-perception system can detect the phase errors, so here we consider the Mach-Zehnder vibro-perception setup as the phase detector. Figure 3 gives the schematic diagram of OPLL vibro-perception system that consists of the Mach-Zehnder interferometer and feedback control loop. The fiber-skin is located in Arm 1 of the Mach-Zehnder interferometer. To increase the fiber-skin sensitivity and decrease the frequency of received signals, we add dithering signals to the phase modulator (PM) from the channel 1 of arbitrary waveform generator (AWG). The amplitude and frequency of dithering signal are 500 mV and 1 MHz. The signals detected from PD mix with a radio frequency (RF) offset with amplitude of 1.5 V and frequency of 1 MHz from the channel 2 of AWG. The mixed signals pass through a 100.8 kHz low-pass filter to remove the high-frequency noises. The core component of the OPLL is the proportional integral derivative (PID) controller or PID filter that is a filter used in feedback control loop. Using the PID filter, the phase errors between Arm 1 and Arm 2 are controlled to zero. In this paper, we use the proportional and integral terms of the PID filter [35]. The transformation function of PID filter is written as follows:

(4) $$F\left(s\right)=K_{p}+\frac{K_{I}}{s}$$

where $K_{p}$ is the proportional gain, and $K_{I}$ is the integral gain. The transfer function of OPLL is written as [36]:

(5) $$\begin{array}{ccc} H(s) & = & \frac{K_{l}K_{p}s+K_{l}K_{I}}{s^{2}+K_{l}K_{p}s+K_{l}K_{I}}\\ & = & \frac{2\zeta \omega_{n}s+\omega_{n}^{2}}{s^{2}+2\zeta \omega_{n}s+\omega_{n}^{2}} \end{array}$$

where $K_{l}$ is the loop gain, and ζ is the damping factor and $\omega_{n}$ is the natural frequency. The relation between ζ, $\omega_{n}$ and gain ($K_{l},K_{p},K_{I}$) are expressed as following equations:

(6) $$\begin{array}{ccc} \zeta & = & \frac{K_{p}}{2}\sqrt{\frac{K_{l}}{K_{I}}} \end{array}$$

(7) $$\begin{array}{ccc} \omega_{n} & = & \sqrt{K_{l}K_{I}} \end{array}$$

Because the response time, the oscillation amplitude and the steady state of the loop are governed by the value of ζ, $\omega_{n}$, we adjust the proportional and integral gain to keep the loop stable.

Since the voltage ranges of the PID (−15 V to +15 V) and piezo driver (PZD) (0 v to 10 v) are different, a signal conditioning (SC) circuit is designed to match the voltage differences. The fiber stretcher (FS) driven by PZD is used as optical phase modulator. To get better beating results, we use the polarization controller (PC) to adjust the light polarization to equalize between the two arms. Moreover, the fiber length between two arms is same (eighteen meters) and the input powers of coupler 2 from the Arm 1 and Arm 2 are identical by tuning the attenuator in the Arm 1.

## 3. Experiment

### 3.1. Experimental Setup

**Figure 4:**
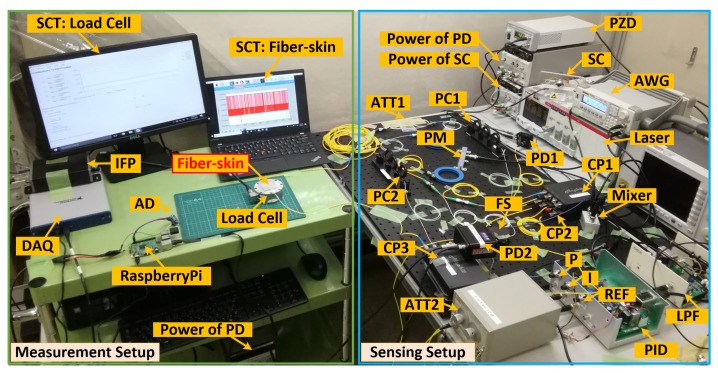
Experimental setup of fiber-skin vibro-perception. The coupler 3 is a 5% coupler that is used to attenuate optical power here. The output power and wavelength of laser are 6.02 dBm and 1543.3 nm. *P*, *I* and REF in the PID box present the proportional, integral and reference knob respectively. ATT2 is the optical attenuator used to control the input power of PD2. CP: coupler.

Presented in this section is the experimental setup of fiber-skin vibro-perception system based on the schematic diagram in Section 2. As shown in Figure 4, the setup, including the measurement and sensing setup, is used to demonstrate the performance of the Mach-Zehnder perception (PID: off) and OPLL perception system (PID: on). The measurement setup consists of the signal collection terminal (SCT) of fiber-skin for data processing and a load cell (F/T Sensor Gamma, ATI) as reference. The load cell is a tri-axis transducer for measuring the force applied on fiber-skin. The interface power (IFP) supply (9105-IFPS-1) is used to power the load cell and the data acquisition (DAQ) card (USB-6341, National Instruments) is used to communicate with SCT of load cell. The signal processing unit (RaspberryPi 3), connected with a 16 bits analog-to-digital (AD) converter (ADS1115, Texas Instruments), is used for signal collection of fiber-skin through the inter-integrated circuit ($I^{2}C$) protocol.

Based on the schematic diagram of vibro-perception system in Figure 3, the sensing setup is constructed by the Mach-Zehnder interferometer and control loop. In the control loop, the low-pass filter (LPF) and PID (T-01BEF01C and T-PID01Z, Turtle Industry) are used to detect the phase errors of optical interference and then provide the feedback to the Arm 2 driven by the PZD (MDT694B, Thorlabs). The PZD voltage ranges from 0 V to 150 V. Because the optical interference intensity is highly sensitive to the feedback signals, we select precision potentiometers (3590S, Bourns) to fine tune the proportional and integral gain of PID. Since the beating results behave as cosine wave, the voltage would be fixed to the middle point (75 V) of PZD by tuning the SC when the PID is switched off. The beating pattern induced by the interferometer are split into two parts by the coupler-2. The signals detected by PD-1 are used for the OPLL feedback. The sensing results of fiber-skin are identified by PD-2.

### 3.2. Experimental Details of Fiber-Skin Vibro-Perception System

We conduct the direct contact and indirect contact experiments to demonstrate the perceptual performance of optical fiber-skin. Table 1 illustrates the experimental details.

**Figure 5:**
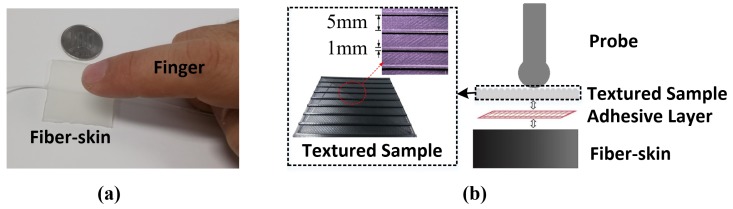
Schematic digram of experimental examples. (**a**) Example of direct contact experiment; (**b**) example of indirect contact experiment.

In the first experiment, we demonstrate the direct contact experiment for both the Mach-Zenhder and OPLL vibro-perception system with the tapping and sliding motions. Figure 5a shows direct contact experiment with the index finger. Prepared fiber-skin sample is used for both direct and indirect contact experiments. For stabilizing the optical perception system, the experimental setup runs prior to the experiment for five minutes.

Another experiment is designed to evaluate the vibro-perception with indirect contact. In this research, a sample (50 mm × 50 mm) with nine ridges and eight grooves is fabricated by the 3D printer (Anycubic I3 MEGA) to demonstrate the perceptual ability of fiber-skin (refer to Figure 5b). The dimension of ridges on the sample are identical, and each one is 1 mm × 50 mm × 0.5 mm (width, length, and height) with a spacing of 5 mm. The textured sample is attached to the fiber-skin by using an adhesive layer (double-sided tape) to avoid unnecessary movement during experiments.

**Figure 6:**
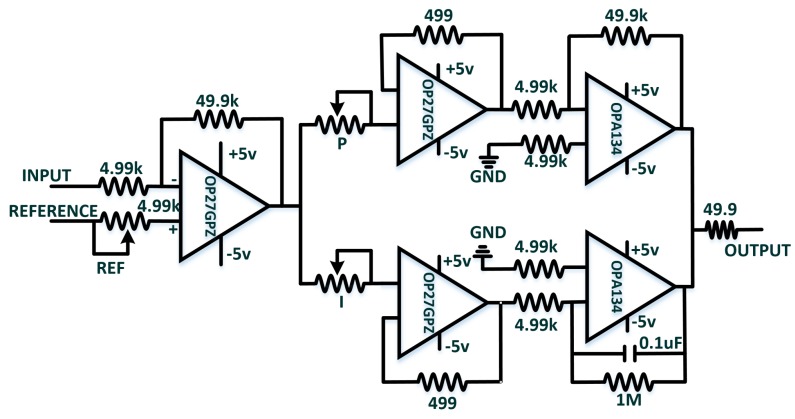
Schematic diagram of PID controller. The differential operational amplifiers, OP27GPZ and OPA134, are used to construct the PID controller. The amplifiers work for the function of buffer, adder and signal integral. *P*, *I* and REF correspond to the knobs of *P*, *I* and REF on the PID controller box. The INPUT signals are from the LPF’s outputs and the REFERENCE port is connected with a 5 v power supply. The OUTPUT is the PID output port.

The essential component in the OPLL perception system is the PID controller that is designed to lock the phase errors to zero. First of all, the PID adjustment method will be described in this section. Figure 6 shows the schematic diagram of PID controller.

The phases between two arms can be locked by turning each knob of proportional (*P*), integral (*I*) and reference (REF) on the PID box. The PID controller consists of the operational amplifier (OP27GPZ and OPA134, Texas Instruments) and external electronic circuits. To search for the steady state of reference signal, the integral parameter is initially set to 0.1 and then increase the scale of proportion parameter until the outputs of PID oscillate. Next, the steady states of the PID will be fixed by fine tune the reference parameter. At this moment, the LPF’s outputs converge to a constant, but they show the tendency of rising or falling when the steady states are attained. The trend depends on the sign (positive or negative) of the difference between the reference and input signals. Finally, the phase errors are locked by increasing the amplitude of *I*. Once the phases are locked, the vibro-perception properties of fiber-skin can be tested by switching off/on the PID controller.

## 4. Results and Discussion

### 4.1. Experimental Results of Mach-Zehnder Vibro-Perception

**Figure 7:**
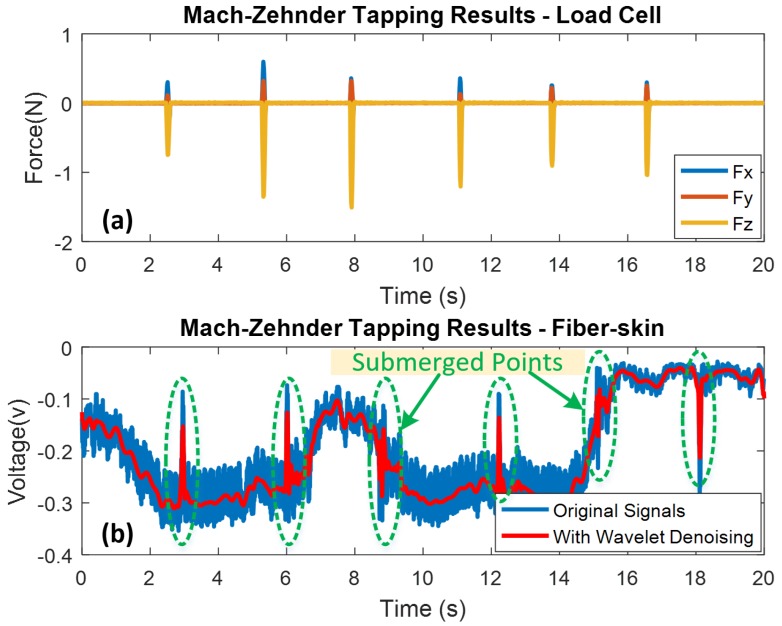
Tapping results of Mach-Zehnder vibro-perception system with high precision tri-axis load cell as reference. Wavelet denoising method is used for high frequency noise elimination. (**a**) Mach-Zehnder tapping results from load cell; (**b**) Mach-Zehnder tapping results from the SCT of fiber-skin.

As mentioned above, the Mach-Zehnder vibro-perception system is an open-loop system, and the beating pattern between two arms behave as cosine wave. In this experiment, the fiber-skin is slightly tapped by the index finger as shown in Figure 5a. The finger stays on the fiber-skin around 0.2 s and releases 3 s until next tapping. For the Mach-Zehnder experiment, the SCT sampling rate of fiber-skin is 860 samples per second, and the data length is 20 s. Because the separate signal collection setups of load cell and fiber-skin, a minor time delay of signals detected by the SCT of load cell and fiber-skin, exists in the following experimental results. Because the power supply of AD converter from the signal processing unit introduces high-frequency noises to the sensing signals, the wavelet denoising method is used to reduce the high-frequency noises. In total, six tapping motions are conducted, accordingly, the same number of periodic peaks of vibration signals are detected by the load cell (refer to Figure 7a). Each peak indicates the tapping motion applied on the fiber-skin simultaneously. Since the finger tapping will introduce disturbance to the Arm 1, the interference intensity (cosine wave) between two arms varies quickly when the tapping occurs. Figure 7b shows that two submerged points are observed because tapping motions are "swallowed" by the interruption. Furthermore, we can observe that the direction of sensing voltage depends on the values of beating results, that is to say, the sensing signal shows an upward spike when the beating results are at the minimum value, and vice versa. However, the sensing signals are submerged in the beating results when the tapping motions are occurred in the middle of beating results.

**Figure 8:**
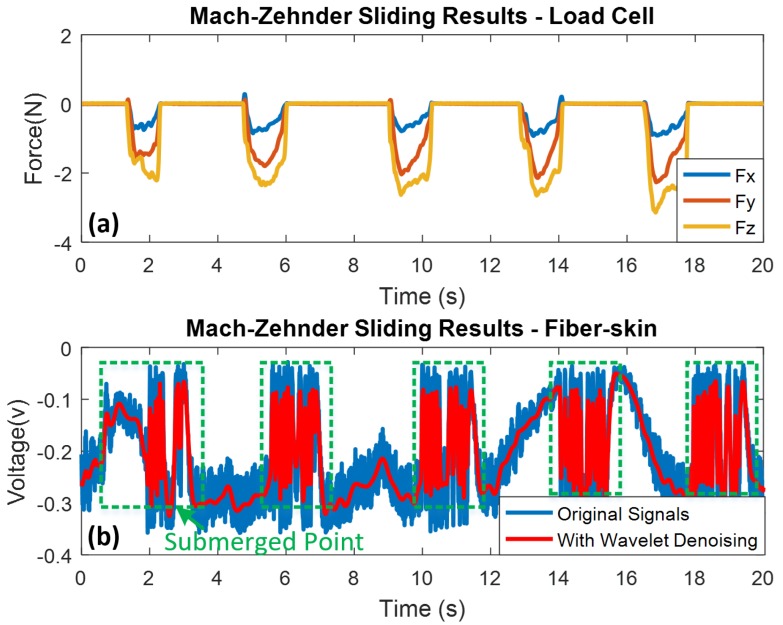
Sliding results of Mach-Zehnder vibro-perception system with high precision tri-axis load cell as reference. Wavelet denoising method is used for high frequency noise elimination. (**a**) Mach-Zehnder sliding results from load cell; (**b**) Mach-Zehnder sliding results from the SCT of fiber-skin.

An additional sliding experiment is carried out to evaluate different perception properties of the optical fiber-skin. The sliding time is 1 s for each motion with 3 s interval. To keep the experimental consistency, the whole sliding time is restrained to 20 s. Five sliding motions with different force (2.00 N to 3.14 N) are detected by the load cell (refer to Figure 8a). After the sliding continuously explores on the fiber-skin, the intensity of sensing signal is gradually reduced. This phenomenon is consistent with dynamic friction theory according to the literature. Morales-Hurtado et al. has investigated the ex-vivo human skin to explain the tactile friction of sliding. The highest coefficient of friction (COF) is observed at the starting point of running-in period, and then dynamic friction decreases to a stable value [37]. As shown in Figure 8b, at the initial moment of sliding motion, the peak signal detected by the SCT of fiber-skin appears to be identical as tapping motion. However, the dynamic response of fiber-skin is saturated with no signal fluctuation.

As mentioned in Equation (3), the detected signals of the fiber-skin behave as cosine waves, therefore, the perception signals are impacted by the beating intensity. Compared with the tapping results, a submerged point disturbed by the beating interference is also observed when the sliding motion occurs in the middle of beating results.

### 4.2. Experimental Results of OPLL Vibro-Perception

In the following section, we will present the tapping and sliding experimental results of the fiber-skin by using the OPLL vibro-perception system. Similar to the experiment of Mach-Zehnder vibro-perception system, the SCT sampling rate of fiber-skin is also 860 samples per second, and the data length is 20 s as well. In this experiment, the contact time between the index finger and fiber-skin is approximately 0.2 s, and the tapping interval is still 3 s.

**Figure 9:**
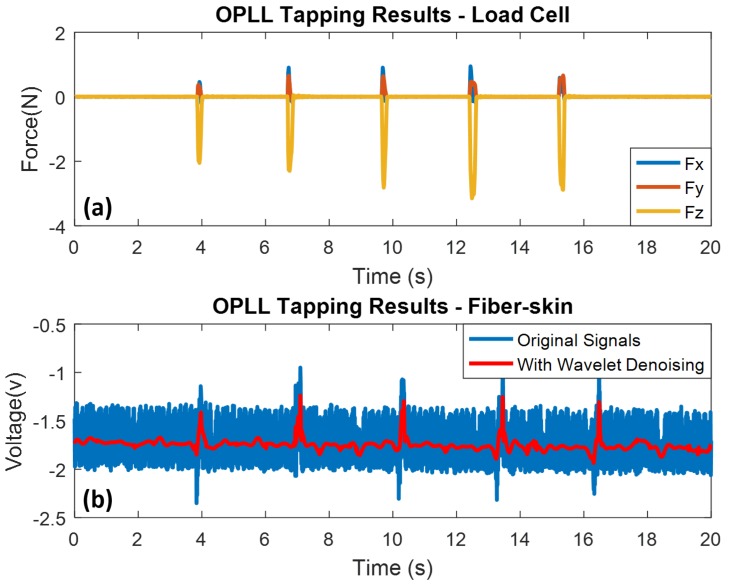
Tapping results of OPLL vibro-perception system with high precision tri-axis load cell as reference. Wavelet denoising method is used for high frequency noise elimination. (**a**) OPLL tapping results from load cell; (**b**) OPLL tapping results from the SCT of fiber-skin.

Apparently, five periodic tapping peaks are detected by the load cell during the time frame of 20 s. As introduced in Section 2, the amplitude of RF offset is 1.5 V, so the sensing voltage of OPLL vibro-perception system is biased by the offset. Like the detected tapping motion by the load cell, five tapping spikes are observed in the results (refer to Figure 9a). The submerged points, disturbed by the beating interference, do not exist in the sensing results because the PID controller provides feedback to the optical path after the tapping occurs. In this case, the high frequency and low amplitude external disturbance can be reduced by the OPLL vibro-perception system.

**Figure 10:**
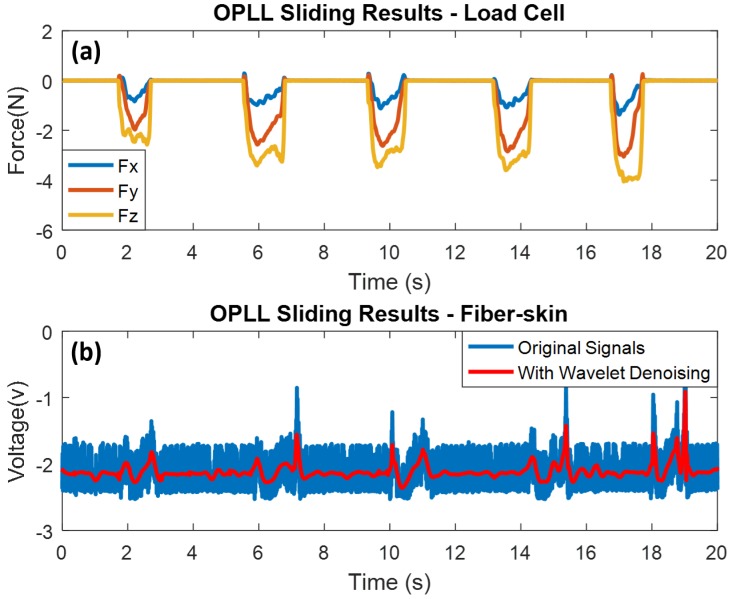
Sliding results of OPLL vibro-perception system with high precision tri-axis load cell as reference. Wavelet denoising method is used for high frequency noise elimination. (**a**) OPLL sliding results from load cell; (**b**) OPLL sliding results from the SCT of fiber-skin.

In this section, the results of the sliding experiment with the OPLL vibro-perception system are presented. The sliding time and interval is 1 s and 3 s in each motion. Five sliding motions are observed from the load cell, and the corresponding sensing voltages are also detected by the fiber-skin (refer to Figure 10a,b).

A spike appears at the beginning of sliding, because the starting point of sliding is also a tapping motion. In addition, it has the highest COF at the starting point with a running-in period, and then the COF decreases to a stable state. Compared with the Mach-Zehnder setup, the submerged points (disturbed by beating interference) are not observed in the sensing results of OPLL vibro-perception system. The load force applied on the fiber-skin ranges from 2.20 N to 4.10 N, and the corresponding sensing voltages ranges from −0.52 V to 0.59 V.

Based on the above results, we can conclude that the Mach-Zehnder vibro-perception system possesses the characteristics of high sensitivity compared with OPLL vibro-perception system in the direct contact experiment. This high sensitivity is consistent with skin perception in light touch regime that is defined by force less than 1 N [38]. However, the Mach-Zehnder system has low susceptibility to beating interference for both tapping and sliding motions. Comparatively, the OPLL system is more robust to the beating noise. In the case of direct contact experiment, OPLL vibro-system has better performance compared to the Mach-Zehnder vibro-system.

### 4.3. Results of Indirect Experiment

For further demonstration of the fiber-skin perceptual performance, the following indirect contact experiment is designed. Yoshioka et al. has investigated texture perception by holding a probe attached with tri-accelerometer to measure the vibration for indirect perception. Based on the experimental results, texture-generated vibrations contain enough information to discriminate perceived roughness [39].

**Figure 11:**
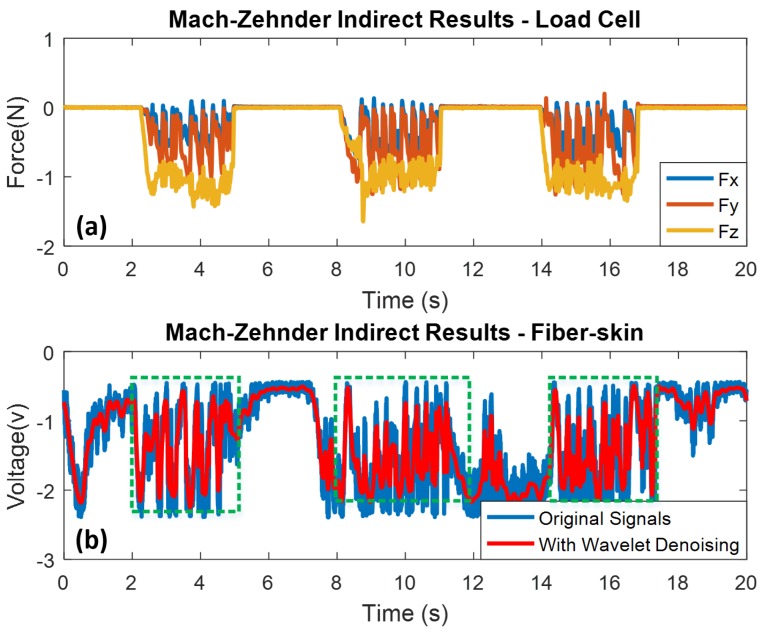
Texture sliding results of Mach-Zehnder vibro-perception system with indirect experiment. Wavelet denoising method is used for high frequency noise elimination. (**a**) Indirect sliding results from load cell; (**b**) indirect sliding results from the SCT of fiber-skin.

**Figure 12:**
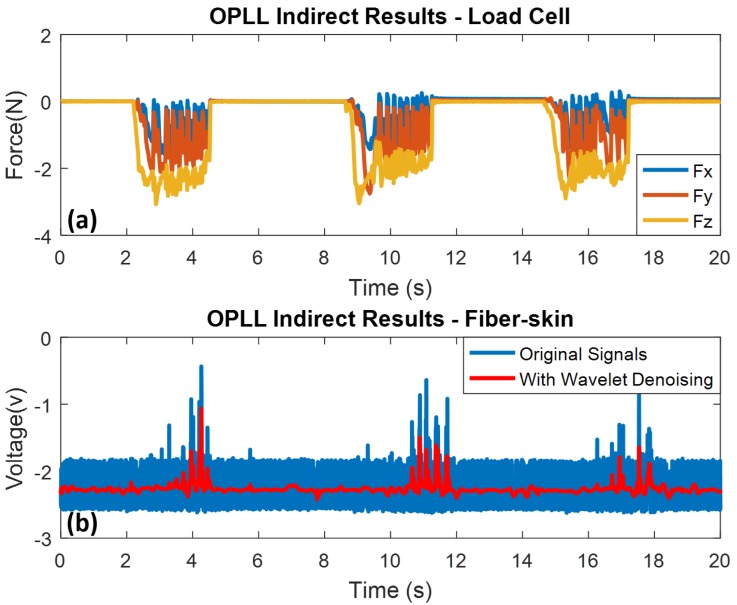
Texture sliding results of OPLL vibro-perception system with indirect experiment. Wavelet denoising method is used for high frequency noise elimination. (**a**) Indirect sliding results from load cell; (**b**) indirect sliding results from the SCT of fiber-skin.

In our research, the fiber-skin demonstrates the same ability as the human skin in perceiving surface texture through indirect perception based on the vibration signals obtained from the fiber skin by using the Mach-Zehnder and OPLL vibro-perception system. As shown in Figure 11a, the indirect sliding time is 3 s and time interval is 3 s as well. The surface pattern on the textured sample consists of eight convex ridges. Three sliding motions with eight noticeable peaks are detected by the SCT of fiber-skin (refer to Figure 11b). Figure 12a,b show the indirect sliding of OPLL setup with three sliding motions from the load cell and the SCT of fiber-skin respectively.

Compared with the Mach-Zehnder vibro-perception system, the OPLL system can partially detect the ridges because of the fast response of PID controller with small disturbance. Table 2 shows the load force applied on the Mach-Zehnder and OPLL vibro-perception system. The load forces on the Mach-Zehnder vary from 0.68 N to 1.64 N, and on OPLL vibro-perception system vary from 1.25 N to 3.04 N. The results indicate that the Mach-Zehnder vibro-perception system with lower load force has better performance than the OPLL vibro-perception system.

## 5. Conclusions

In summary, the vibro-perception system of bio-inspired fiber-skin demonstrates the ability to detect the physical motions like tapping and sliding in both Mach-Zehnder (open-loop) and OPLL (closed-loop) perception systems. Both direct and indirect vibro-perception are conducted to validate the ability of fiber-skin to perform as the human skin. The distinguishable signals between tapping and sliding enable the fiber-skin to detect and discriminate the physical motions applied on it as the real skin. Furthermore, the fiber-skin possesses good performance for direct contact experiment with OPLL vibro-perception system while the Mach-Zehnder is preferable to the OPLL system in the indirect contact experiment. Additionally, the fiber-skin has the characteristic of light touch and high repeatability, which is highly-suitable for robotic sensing.

## Figures and Tables

**Table 1:** Experimental details of fiber-skin vibro-perception system.

Types	Direct Contact	Indirect Contact
Mach-Zehnder	Tapping & Sliding	Texture Sliding
OPLL	Tapping & Sliding	Texture Sliding

**Table 2:** Load force for the indirect experiment of Mach-Zehnder vibro-perception and OPLL vibro-perception system.

Types	No.	Maximum (N)	Minimum (N)	Mean (N)
Mach-Zehnder	1	1.43	0.95	1.16
	2	1.64	0.73	1.09
	3	1.35	0.68	0.92
OPLL	1	3.01	1.90	2.32
	2	3.04	1.25	2.06
	3	2.90	1.56	2.18
